# Multi-modular AI Approach to Streamline Autism Diagnosis in Young Children

**DOI:** 10.1038/s41598-020-61213-w

**Published:** 2020-03-19

**Authors:** Halim Abbas, Ford Garberson, Stuart Liu-Mayo, Eric Glover, Dennis P. Wall

**Affiliations:** 1 Cognoa Inc., Palo Alto, CA USA; 2 Departments of Pediatrics, Biomedical Data Science and Psychiatry and Behavioral Sciences, Stanford University, Stanford, CA USA

**Keywords:** Human behaviour, Autism spectrum disorders

## Abstract

Autism has become a pressing healthcare challenge. The instruments used to aid diagnosis are time and labor expensive and require trained clinicians to administer, leading to long wait times for at-risk children. We present a multi-modular, machine learning-based assessment of autism comprising three complementary modules for a unified outcome of diagnostic-grade reliability: A 4-minute, parent-report questionnaire delivered via a mobile app, a list of key behaviors identified from 2-minute, semi-structured home videos of children, and a 2-minute questionnaire presented to the clinician at the time of clinical assessment. We demonstrate the assessment reliability in a blinded, multi-site clinical study on children 18-72 months of age (n = 375) in the United States. It outperforms baseline screeners administered to children by 0.35 (90% CI: 0.26 to 0.43) in AUC and 0.69 (90% CI: 0.58 to 0.81) in specificity when operating at 90% sensitivity. Compared to the baseline screeners evaluated on children less than 48 months of age, our assessment outperforms the most accurate by 0.18 (90% CI: 0.08 to 0.29 at 90%) in AUC and 0.30 (90% CI: 0.11 to 0.50) in specificity when operating at 90% sensitivity.

## Introduction

Idiopathic forms of Autism Spectrum Disorder (ASD) have no known biological cause and may correspond to multiple conditions with similar symptoms. The incidence of ASD has increased in recent years, and it impacts 1 in 59 children according to the latest studies^1^. ASD is diagnosed from clinical observations according to standard criteria^2^ relating to the child’s social and behavioral symptoms. Autism is said to be on a spectrum due to the varied severities of symptoms, ranging from relatively mild social impairment to debilitating intellectual disabilities, inabilities to change routines and severe sensory reactions^2^. Approximately 25–50%^3^ of autistic children are non-verbal and have severe symptoms.

Notably, diagnosis within the first few years of life dramatically improves the outlook of children with autism, as it allows for treatment during a key window of developmental plasticity^4,5^. Unfortunately, the latest studies show that although 85% of parents of children with autism reported developmental concerns about their children by 36 months of age, the median age of diagnosis in the United States is 52 months^1^. The complexity of the diagnostic procedures and the shortage of trained specialists can result in children with ASD not getting a diagnosis early enough to receive behavioral therapies during the time when they are most effective.

Diagnosing autism in the United States generally takes two steps: developmental screening followed by comprehensive diagnostic evaluation if screened positive^6^. Screening instruments typically use questionnaires that are answered by a parent, teacher or clinician^7,8^. They are generally easy and inexpensive to administer and can be useful to flag some at-risk children, however, they are not always accurate enough to help inform a diagnosis^9^. Standard autism screeners can also have a high false positive rate, leading to unnecessary referrals and healthcare costs^10^. Comprehensive diagnostic evaluation instruments, on the other hand, are more accurate but require long and expensive interactions with highly trained clinicians^11,12^.

In this paper, we present improvements to two previously published^13^ automated autism assessment modules underlying the Cognoa^14^ software. The first module is based on a brief questionnaire about the child presented directly to parents without supervision. The second module is based on lightly trained analysts evaluating short videos of children within their natural environment that are captured by parents using a mobile device. We also present a new, third module that is intended to be completed in a primary care setting such as a pediatrician’s office during a clinic visit. The third module is based upon a questionnaire that is answered by a clinician after examining the child and talking to the parent. We demonstrate that these three modules are as fast and easy to administer as most of the typical screening instruments, yet their combined assessment accuracy is shown in this work to be significantly higher, such that they may be used to aid in diagnosis of autism.

We present our approach to selecting maximally predictive features for each of the modules. Both the parent and the clinician questionnaire modules key on behavioral patterns similar to those probed by a standard autism diagnostic instrument, the Autism Diagnostic Interview - Revised (ADI-R)^11^. ADI-R is administered by a trained clinician, and typically gives consistent results across examiners. But its 93 point questionnaire often spanning 2.5 hours of the interviewer and parent’s time makes it largely impractical for the primary care setting^15^. The video assessment module keys on behavioral patterns similar to those probed in another diagnostic instrument, the Autism Diagnostic Observation Schedule (ADOS)^12^. ADOS is a multi-modular diagnostic instrument, with different modules for subjects at different levels of cognitive development. It is widely considered a gold standard and is one of the most common behavioral instruments used to aid in the diagnosis of autism^16^. It consists of an interactive and structured examination of the child by trained clinicians in a tightly controlled setting.

For validation, the three modules are applied to assess children in a clinical study using the Cognoa^14^ software. To-date, Cognoa has been used by over 300,000 parents in the US and internationally. The majority of Cognoa users are parents of young children between 18 and 48 months. The clinical study underlying the validation results discussed in the results section consists of a total of 375 at-risk children who had undergone full clinical examination and received a clinical diagnosis at a center specialized in neurodevelopmental disorders^17^. The outputs of the assessment modules are compared to those of three screening instruments. The Modified Checklist for Autism in Toddlers, Revised (M-CHAT-R)^7^ is a parent-completed questionnaire for autism that is intended to be administered during developmental screenings for children between the ages of 16 and 30 months and is commonly used as an autism screening instrument. The Social Responsiveness Scale - Second Edition (SRS) is another standard ASD screener that is based upon a questionnaire filled out by an examiner^18–20^. The SRS has a preschool form intended for children of ages 30 months to 54 months, and a school age form intended for children of ages 48 months through 18 years of age. We use SRS “total score” scale as a baseline autism assessment. The Child Behavior Checklist (CBCL)^8^ is a parent-completed questionnaire that provides risk assessments in many categories. We use the “Autism Spectrum Problems” scale of CBCL for comparison. In all cases, the answers to the questions comprising the screeners are coded, then the codes are summed and the sum compared against a threshold to determine whether the child is at risk.

## Methods

We base our approach on de-identified historical patient records. We collect medical instrument score sheet data pertaining to children tested for suspicion of autism, and process those into training sets for the predictive models underlying each of our three autism assessment modules.

Since we apply said predictive models in a significantly different setting than the clinics where the corresponding training data were generated, we expect a consequential performance degradation resulting in unacceptable diagnostic accuracy if conventional machine learning methods are used^13^. To counteract that effect, we apply custom machine learning techniques as detailed in this section, building upon previous experimental work^13^. The new techniques discussed below are empirical post-hoc feature selection, training data noise injection, and an overfitting-resilient probabilistic combination of module outcomes.

### Data

Training data were compiled from multiple repositories of de-identified ADOS and ADI-R score sheets of children between 18 and 84 months of age including Boston Autism Consortium, Autism Genetic Resource Exchange, Autism Treatment Network, Simons Simplex Collection, and Vanderbilt Medical Center. To counteract class imbalance, the sample set negative class was supplemented with 59 low risk children random-sampled from Cognoa’s user-base, and ADI-R was administered on those additional controls.

**Figure 1:**
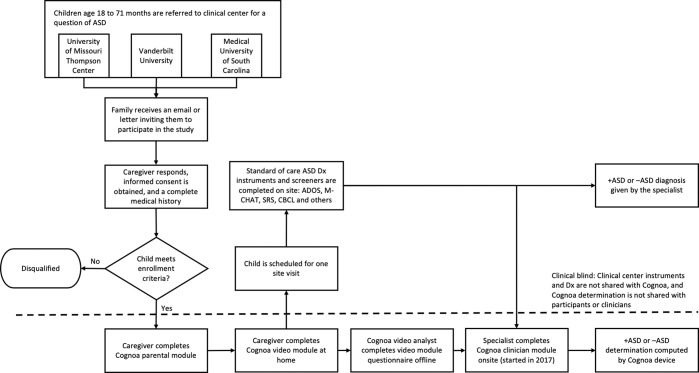
Detailed steps performed during the clinical study described in this document.

The diagnostic accuracy of our modules was measured using data from a multi-site blinded clinical validation study (reviewed and approved by Western IRB project number 2202803)^17^. The study was performed in 2016 and 2017 at three tertiary care centers in the United States. Informed consent was obtained from guardians of each child, and all relevant regulations and guidelines were followed. Children enrolled in the study were 18 to 72 months of age, of English-speaking households, and were all referred through the typical referral process for suspicion of autism. Every child was measured using autism assessment instruments (such as ADOS, M-CHAT-R, and/or CBCL) as appropriate for his or her age. Diagnosis was ultimately ascertained by a licensed health care provider. Prior to the clinical assessment, parents used the Cognoa mobile app to complete the parent questionnaire and video assessment modules, and starting in 2017, a clinician also completed the Cognoa clinician questionnaire. The clinicians were blinded to the results of the assessment rendered by Cognoa. More details on the steps of the clinical study are shown in Fig. 1.

The enrollment process in 2016 yielded 162 validation samples, which were used to validate the parent questionnaire and video modules. This same clinical enrollment cohort was used as validation dataset in our previous publication on the subject^13^. Given the learnings from this dataset, and prior to the extension of the study in 2017, several improvements were made to the algorithms including tuning of model thresholds, training combination modules, and performing feature selection for the clinician module which was newly introduced in 2017. The enrollment process in 2017 yielded 213 additional validation participants, bringing the total N to 375 samples over the course of the two years.

The sample breakdown by cohort, age group, and diagnosis for all data used for training and validation is shown in Table 1. In both the training and the validation datasets, the majority of the “Not autism” class label is composed mostly of children who are diagnosed with an alternate developmental delay (e.g., ADHD or speech and language disorder). Since these conditions share many symptoms with autism, this is a particularly challenging sample for classification . Only seven of the children in the validation sample are neurotypical, suggesting that this sample will be harder to perform correct classifications on than in the general population.

**Table 1:** Dataset breakdown by age group and condition for each of the sources of training data and for the clinical validation sample. Machine learning model training was stratified by age group. Clinical validation 2016 and 2017 samples are used together to evaluate performance of the parent and video modules in this paper, while the clinician module was only available for the clinical 2017 dataset.

Age (years)	Condition	Number of samples			
		Parent/Clinician module training	Video module training	Clinical validation 2016	Clinical validation 2017
<4	Autism	414	1445	75	91
<4	Not autism	207	539	20	30
≥4	Autism	1885	1865	46	60
≥4	Not autism	180	410	21	32

### Algorithm methodology

In this section we explain important aspects of our machine learning methodology that are common to the classifiers underlying each of our three assessment modules.

#### Training procedure

Classifier training, feature selection, and optimization were done separately for children under four years of age and four years of age and over. The parent questionnaire and clinician questionnaire classifiers make predictions based off of answers to questions that probe similar concepts to the ADI-R questionnaire. They were trained using the answers to questions from historical item-level ADI-R score sheets with labels corresponding to established clinical diagnoses. The video module makes predictions based off of answers to questions that probe similar concepts to the ADOS instrument, as recorded by video analysts. It was trained using ADOS instrument score sheets and diagnostic labels. Progressive sampling was used to verify sufficient training volume as detailed in the supplementary materials. Gradient boosted decision trees were used for all three modules as they consistently performed better than other options that were considered such as neural networks, support vector machines, and logistic regression. For all models, hyper-parameters were tuned with a bootstrapped grid search. In all cases, true class labels (ASD or non-ASD) were used to stratify the folds, and (age, label) pairs were used to weight-balance the samples. More details can be found in the supplementary materials.

In all cases, the machine learning models were trained using historical patient records that correspond to controlled clinical examinations, but focused on application in non-clinical settings aimed for brevity, ease-of-use, and/or unsupervised parent usage at home. These differences introduce biases which can be significant enough to ruin the performance of an algorithm if not properly addressed, and which cannot be probed by cross validation. See the supplementary material for further details. New strategies to address these biases are discussed below that result in big improvements in accuracy compared to previous work^13^.

#### Inconclusive outcomes

**Figure 2:**
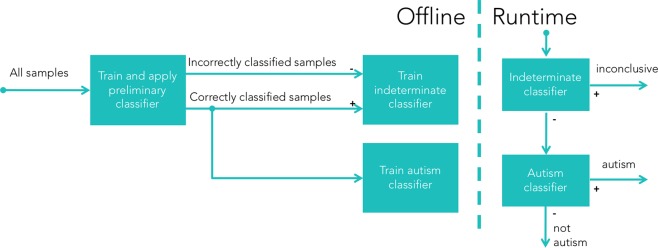
An illustration of the methodology for training diagnostic assessment algorithms capable of outputting one of three possible outcomes: “positive”, “negative”, or “inconclusive”. The first binary classifier is only used to assist in training and never at runtime. It is trained to predict binary “autism” vs “not autism”, and these labels are then compared with the true ASD results to label which samples are incorrectly classified. The samples with their “correct” and “incorrect” labels are used to train the classifiers at runtime. A “indeterminate” classifier is trained to predict which samples will have their ASD diagnosis misclassified, which serves as a filter to identify “inconclusive” cases at runtime, while only the predicted “correct” samples are used to train the final binary ASD diagnosis classifier.

Each of the three modules predicts one of three assessment outcomes: Positive, negative, and inconclusive. As outlined in Fig. 2, support for inconclusive determination is incorporated using a process that involves three separate machine learning training runs. The first model is trained to make predictions that are used to label the samples in the training data that are the most likely to be misclassified. A second model is then trained using these labels to predict the likelihood of any new samples being misclassified. Finally, only samples that are likely to be classified correctly are used to train a final, binary autism classifier. Only the latter two models are used at prediction time: the one to identify and filter out samples that should be labeled “inconclusive”, and the other to make a binary prediction of whether the child is autistic in those which are likely to be correctly labeled. More details about how these models are trained are available in the supplementary material.

### Parental module

#### Initial feature selection

The parental questionnaire probes for a minimal set of highly relevant child behavioral patterns that are maximally predictive of autism in combination. Care is taken to phrase the questions and answers such that the most reliable signal can be input from everyday parents undertaking the questionnaire via mobile app without clinical assistance.

To that effect, a custom feature selection method is devised involving robust bootstrap-driven backwards subtraction, the details of which are discussed in a previous publication^13^. Out of an initial set of 93 questions under consideration, this produces an optimal set of 17 novel questions for children less than four years old, and 21 questions for children four and older.

#### Empirical post-hoc feature selection refinement

Following the conclusion of the 2016 clinical validation study enrollment, we studied differences in the distribution of answers to each question between the training data and the validation data that was collected in the 2016 clinical study. While some questions had quite good agreement, others show a strong bias towards higher (or lower) severity answer choices in the clinical data than in the training data. Questions for which the mean absolute severity difference was statistically greater than three standard errors (averaged over the autism and the non-autism samples) are rejected. This requirement results in the exclusion of 4 out of the 17 questions in the younger cohort, and 8 out of 21 questions in the older cohort, and the models are re-trained (with new hyper-parameter tuning) on the reduced feature set. This further refinement of the selected features minimizes the significant biases due to differences between the training and application environment. See the supplementary material for more details on these differences.

This feature refinement leads to a larger boost in performance compared with^13^ than any other improvement. The size of the performance improvement is validated on the held-out sample of children collected during 2017, where the new models show a statistically equivalent increase in performance compared with the 2016 sample.

### Video module

The video assessment module consists of a parent upload of 2 or 3 mobile videos, each 1 to 2 minutes in length, of the child during play or meal time at home. The underlying algorithm produces autism assessments based upon the responses of at least three minimally-trained analysts who watch the videos and then respond to a behavioral questionnaire.

The data available for training the video module’s classification model are taken from ADOS sessions administered by clinicians in standardized clinical settings. Gradient boosted decision trees are trained keying off of the features identified in the analysis of ADOS records. The questionnaires that the video analysts answer are then created to probe for similar behavioral features as those observed in the training data. A challenge of this methodology is that the module must make predictions in the face of missing features that are not observable in the short videos uploaded by the parents. The video analysts are allowed to skip any questions if not answerable based on the posted videos. On average, analysts skipped questions 15% of the time, with big variations among particular questions. This effect, combined with the large discrepancy in the observation time window from the original clinical examination to the brief home-video version, would result in a big assessment-accuracy degradation unless steps are taken to correct for the bias and variance .

We tackle this problem by introducing bias and variance to the training data in a manner designed to make it statistically similar to the video analyst answers on which the assessments will be run. The data from the 2016 clinical study^13^ is used to develop this methodology, and the performance of the algorithm on the data from the children enrolled in 2017 is used to validate the generalizability of the improvements. Most children who participated in the clinical study are also administered a full ADOS, which provided paired ADOS and video data that we use to determine what noise patterns to add. Using these paired data, we construct a probability map for each question-response set describing the ways video analysts are likely to respond for a given “true” ADOS response. We then use the mapping as a stochastic transform to build a new training data set that can be thought of as the results of a hypothetical experiment in which the technicians watch parent-supplied video of the children in the training data and respond accordingly.

The addition of simulated “setting noise” to the classifier training data leads to a larger boost in performance compared with^13^ than any other improvement^13^. Additionally, the optimal parameters for the resulting decision tree models favor larger tree depth. This is as expected, since the new models are expected to make determinations as to which features are reliable when present, as well as which features to fall back on when the best features are missing.

### Clinician module

We introduce a module to screen for autism using questionnaire responses from a clinician. A pediatrician might answer these questions during a regular checkup. The questions for the clinician were selected in a similar manner as used for the Parental Module (see the supplementary material for details). Responses from both the parent and the clinician are used in a machine learning module in the same manner as described for the parental questionnaire above. Some key behaviors are probed via questions directed at both the parent and the clinician, but the clinician questions are more nuanced and allow for more subtle answer choices. In cases where the parent and the clinician give contradictory answers to the same question, the clinician’s answer overrides that of the parent. The clinician module was introduced to the clinical validation study beginning in 2017. Its results are therefore based on a smaller sample size than those of the other modules.

#### Feature selection

In order to create a brief clinician questionnaire appropriate for the primary care setting, multiple lists of candidate questions are each compiled and ordered using different strategies. The lists are then intersected and prioritized, then the top features in the intersection set are shortlisted. The first list of candidate questions is prepared by considering those questions from the original medical instruments that had been excluded from the parental questionnaire because they were deemed too difficult for a parent to answer reliably. This list is ranked by feature importance values as measured and ranked by a dedicated offline machine learning training and cross validation run in the same manner as performed for initial parental module feature selection. The second list is prepared from the parental questionnaire questions by simulating the effects of parents over or underestimating answer severities on children with machine learning responses near a decision threshold. Children in the training data for whom the model response was between [0 and 0.1] above the ASD-vs-non ASD decision threshold had their question severities dropped one at a time by one severity value, while children who were between [0 and 0.1] below the decision threshold had their question severities raised by one severity category. The questions in this list are then ranked based upon the average size of the resulting shift in model responses. The procedure is repeated for children in the training data between [0.1 and 0.3] above or below the decision threshold. In each case the top 7 questions are selected (with significant overlap). This results to a total of 9 candidate questions for young children and 10 for older children. The third list is prepared by consulting domain experts for an assessment of the likelihood of each candidate question to benefit from a clinician’s input as a complement to the parent’s input. This method is conducted separately for each of the two age-silo groups, and results in an overall clinician questionnaire of 13 questions for children 18 through 47 month old, and 15 questions for children 4 to six years old.

### Module combination

Due to limitations on available training data, it is not possible to train a single combined model that uses the input features from each of the parental, video, and clinician modules. Instead, responses from the modules are each considered to be a probability and combined mathematically^21^ using the equation:

1 $${r}_{comb}=({I}^{T}{\Sigma }^{-1}R)\ast {({I}^{T}{\Sigma }^{-1}I)}^{-1}$$

Where *r*_*comb*_ is the result of the combination, *I* is a vector of 1s, *R* is a vector of responses for each module to be combined, and Σ is the covariance matrix of the response residuals compared to the true diagnosis. The “training” of the combination module consists of calculating the values of Σ to use in this equation, which is done using the responses of each module on data from the clinical study. For each child, the Σ values in the *r*_*comb*_ equation were calculated with that child excluded. This process is similar to leave-one-out cross validation, and ensures that the results reported for our combination procedure do not suffer from overfitting.

Since Eq. (1) produces only a single model response, the determination of “inconclusive” outcomes proceeds in a different manner than for the individual assessment modules. Both a lower and an upper threshold are applied on the combined response. Children with a response less than both thresholds are considered to be non-ASD, children with a response in between the two thresholds are considered to be inconclusive, and children with a response greater than both thresholds are considered to have ASD. As in the single model cases, the two thresholds can be tuned independently to optimize the sensitivity, specificity, and model coverage.

## Results

Each of the individual Cognoa assessment modules, their combinations, as well as 3 baselines based on commonly-used autism screening instruments (CBCL, M-CHAT-R, and SRS) are evaluated on the data collected during a blinded clinical study. When the inconclusive determination feature is turned off and all samples are required to be assessed conclusively, the Cognoa assessment modules achieve ROC AUC up to 0.83 and sensitivity and specificity up to 80% and 75% respectively. Turning on the inconclusive determination feature with an allowance of up to 30% inconclusive outcomes results in an accuracy improvement over the conclusive samples, with AUC up to 0.92 with sensitivity and specificity up to 90% and 83% respectively. This performance is shown to be a statistically significant improvement over each of the baselines used for comparison.

**Figure 3:**
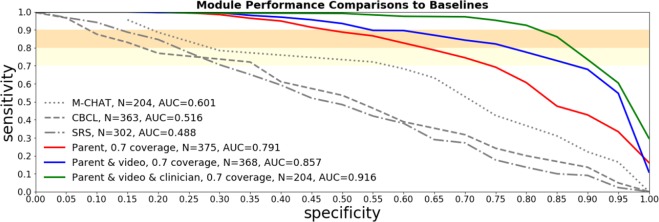
ROC curves on the clinical sample for the parent, video, and clinician modules, separately and in combination. Inconclusive determination is allowed for up to 30% of the cases. The established screening tools M-CHAT-R, SRS-2 and CBCL are compared as baselines. The ROC curve for the M-CHAT-R baseline instrument only includes children under four years of age since M-CHAT-R is not applicable for older children.

**Figure 4:**
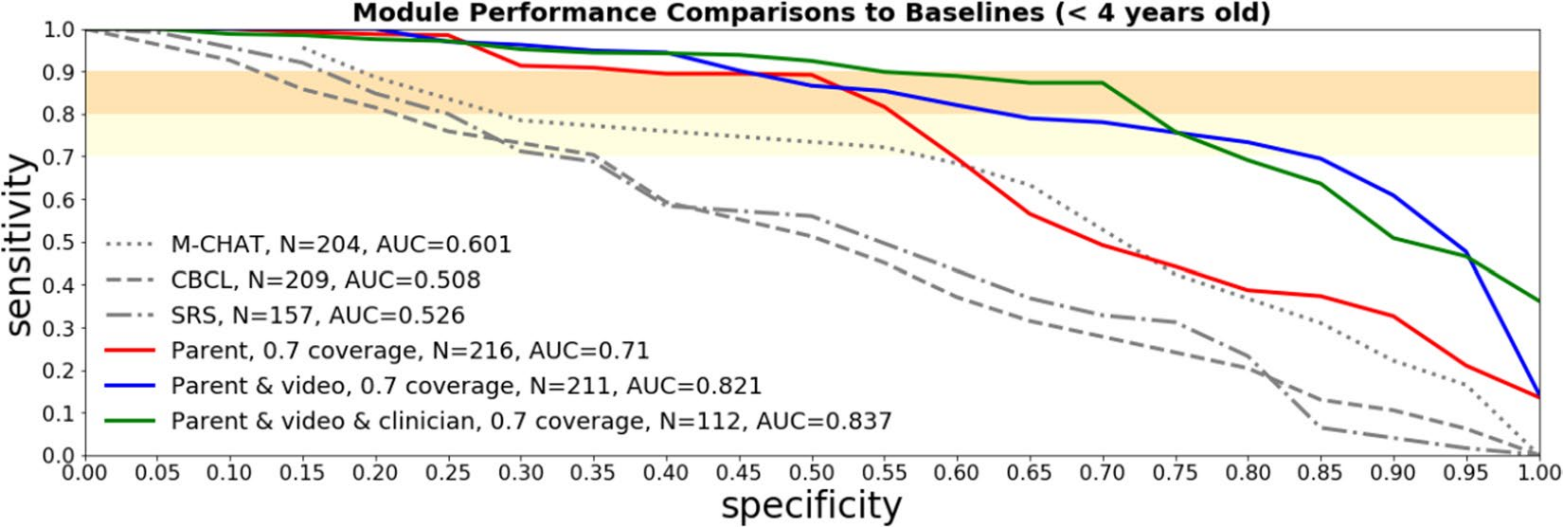
ROC curves on kids  < 4 years of age in the clinical sample for the parent, video, and clinician modules, separately and in combination. Inconclusive determination is allowed for up to 30% of the cases. The established screening tools M-CHAT-R, SRS-2 and CBCL are compared as baselines.

ROC curves in Fig. 3 show how the parent module performs individually, as well as in combination with the video and clinician modules at a 30% inconclusive rate allowance. Figure 4 shows a similar comparison with all the variants consistently restricted to children under four years of age. ROC curves corresponding to the assessment modules with the inconclusive allowance turned off can be found in the supplementary material.

Statistical model performance comparisons between assessment modules and baselines are shown in Table 2. For each comparison, the subset of children for whom both screeners were administered are selected (*n* in the table), and 10,000 bootstrapping experiments are run where *n* children are selected with replacement. The average and [5%, 95%] confidence interval improvements in AUC and the specificity between the screeners are evaluated across all bootstrapping experiments. In the case of specificity the calculation of the improvement is performed using thresholds that are set to achieve 90% sensitivity.

**Table 2:** Statistical tests of performance improvements between models in this paper and standard baseline screening models. ΔAUC tells us the increase in AUC found in the screeners of this paper across bootstrapping experiments. ΔSpecificity tells us the increase in the specificity in the bootstrapping experiments at a threshold designed to achieve 90% sensitivity. Each Δ calculation shows the average value of the improvement along with the [0.05, 0.95] confidence interval.

Age Group	Baseline Screener	Assessment Module	ΔAUC, [0.05, 0.95] C.I.	ΔSpecificity at 90% sensitivity, [0.05, 0.95] C.I.	*n*
All ages	CBCL	Parent	0.17, [0.10, 0.23]	0.21, [0.13, 0.30]	370
All ages	SRS-2	Parent	0.20, [0.12, 0.28]	0.21, [0.12, 0.31]	307
All ages	CBCL	Parent, Video	0.29, [0.22, 0.36]	0.41, [0.30, 0.52]	363
All ages	SRS-2	Parent, Video	0.32, [0.24, 0.40]	0.41, [0.30, 0.52]	302
All ages	CBCL	Parent, Video, and Clinician	0.35, [0.26, 0.43]	0.69, [0.58, 0.81]	200
All ages	SRS-2	Parent, Video, and Clinician	0.42, [0.33, 0.50]	0.65, [0.52, 0.78]	175
1.5 to 3 y.o.	M-CHAT-R	Parent	−0.01, [−0.10, 0.07]	0.09, [−0.02, 0.21]	209
1.5 to 3 y.o.	CBCL	Parent	0.12, [0.03, 0.22]	0.20, [0.07, 0.34]	214
1.5 to 3 y.o.	SRS-2	Parent	0.15, [0.03, 0.27]	0.24, [0.12, 0.38]	161
1.5 to 3 y.o.	M-CHAT-R	Parent, Video	0.14, [0.06, 0.22]	0.20, [0.07, 0.34]	204
1.5 to 3 y.o.	CBCL	Parent, Video	0.28, [0.18, 0.38]	0.33, [0.18, 0.49]	209
1.5 to 3 y.o.	SRS-2	Parent, Video	0.31, [0.20, 0.42]	0.37, [0.21, 0.55]	157
1.5 to 3 y.o.	M-CHAT-R	Parent, Video, and Clinician	0.18, [0.08, 0.29]	0.30, [0.11, 0.50]	107
1.5 to 3 y.o.	CBCL	Parent, Video, and Clinician	0.34, [0.22, 0.45]	0.46, [0.25, 0.67]	111
1.5 to 3 y.o.	SRS-2	Parent, Video, and Clinician	0.40, [0.27, 0.53]	0.50, [0.28, 0.73]	91
1.5 to 3 y.o.	CBCL	Parent	0.21, [0.11, 0.30]	0.23, [0.11, 0.35]	156
1.5 to 3 y.o.	SRS-2	Parent	0.25, [0.15, 0.36]	0.18, [0.06, 0.31]	146
4 to 6 y.o.	CBCL	Parent, Video	0.30, [0.20, 0.39]	0.49, [0.34, 0.64]	154
4 to 6 y.o.	SRS-2	Parent, Video	0.33, [0.22, 0.44]	0.44, [0.29, 0.59]	145
4 to 6 y.o.	CBCL	Parent, Video, and Clinician	0.35, [0.23, 0.47]	0.93, [0.83, 1.00]	89
4 to 6 y.o.	SRS-2	Parent, Video, and Clinician	0.43, [0.30, 0.55]	0.79, [0.66, 0.91]	84

Table 2 shows that Cognoa modules show an improvement of at least 0.26 in AUC and at least 0.52 in specificity compared with the CBCL and SRS-2 screeners at 95% confidence level. Due to the fact that M-CHAT-R screener is only evaluated on younger children the statistical uncertainty in the comparison is larger, however, it also shows an improvement of at least 0.08 in AUC and 0.11 in specificity at 95% confidence level. In these comparisons we allow Cognoa assessment modules to decide to hold aside up to 30% of the hardest cases as inconclusive. The same comparisons when we force the classification on all of the hardest cases can be found in Table 3 of the supplementary material.

### Time to completion comparisons

A random sample of 529 Cognoa users was used in order to measure time to completion of each of the Cognoa autism assessment modules. The median time to completion of the parent module was just under 4 minutes. The median time to completion of the clinician module was 1.2 minutes. The median time per video analysts to score a videos was 20 minutes. More details can be found in the supplementary material. The results indicate that the parent and clinician modules can be completed in as little time as most established autism screeners and in some cases much faster, while achieving significantly higher accuracy. The time required for a video analyst to score a video is more lengthy, however, the turnaround time is faster than for an ADOS administration^12^ and can be performed by minimally trained analysts as opposed to certified clinical practitioners.

## Discussion

We presented a multi-modular assessment consisting of three machine learning modules for the identification of autism via mobile App as well as an evaluation of their performance and time-to-completion in a blinded clinical study. The assessment modules outperform conventional autism screeners, as shown in Table 2 and Fig. 3. The accuracy of the combined assessment is similar to that of gold-standard instruments such as ADOS and ADI-R^22^, without requiring hours of time from certified clinical practitioners. This suggests the potential for the Cognoa assessment to be useful as an autism diagnostic. The high performance of these modules benefits from the use of the techniques described in this paper to identify and set aside up to 30% of the most challenging samples as inconclusive. The supplementary material of this paper shows that we outperform conventional autism screeners without this technique as well.

Important open questions remain. First, in all cases in this paper, the assessment modules were validated on children who had been preselected as having high risk of autism. Children that are pre-selected in this way tend to have autism-like characteristics regardless of their true diagnosis, increasing the challenge of distinguishing true ASD cases. These modules are expected to perform better on a general population sample of children. Further work is needed to verify this hypothesis by conducting clinical studies on children from the general population. Second, the clinician module newly presented in this work appears promising, but so far it has only been applied in a secondary-care setting. Further testing in primary care clinics is needed to validate accuracy in that setting. In addition, two wider avenues of exploration are interesting as further steps. First, while these assessment modules have been shown to be effective at identifying the presence or absence of autism, our goal is to extend them to identify the severity of the condition (if present) as well. Second, the techniques presented in this paper could potentially be used to build algorithms for other child behavioral conditions than autism, as well as behavioral conditions affecting adults and seniors.

## Supplementary information

Supplementary Information.

## References

[1] Baio J (2018). Prevalence of autism spectrum disorder among children aged 8 years— autism and developmental disabilities monitoring network, 11 sites, united states, 2014. MMWR Surveill Summ.

[2] Association., A. P. & Association., A. P. *Diagnostic and statistical manual of mental disorders : DSM-5* (American Psychiatric Association Arlington, VA, 2013), 5th ed. edn.

[3] Patten, E., Ausderau, K. K., Watson, L. R. & Baranek, G. T. Sensory response patterns in nonverbal children with asd. *Autism Res. Treat*.**2013**, 10.1155/2013/436286 (2013). 10.1155/2013/436286 PMC3727194 23956859

[4] Dawson G, Bernier R (2013). A quarter century of progress on the early detection and treatment of autism spectrum disorder. Dev. Psychopathol..

[5] Dawson, G. *et al*. Randomized, controlled trial of an intervention for toddlers with autism: The early start denver model. *Pediatrics* **125**, e17–e23 10.1542/peds.2009-0958, http://pediatrics.aappublications.org/content/125/1/e17.full.pdf (2010). 10.1542/peds.2009-0958 PMC4951085 19948568

[6] Gordon-Lipkin, E., Foster, J. & Peacock, G. Whittling down the wait time exploring models to minimize the delay from initial concern to diagnosis and treatment of autism spectrum disorder. *Pediatr. clinics North Am*.**63**, 851–859 10.1016/j.pcl.2016.06.007 (2016). Exported from https://app.dimensions.ai on 2018/10/19. 10.1016/j.pcl.2016.06.007 PMC5583718 27565363

[7] Bernier R, Mao A, Yen J (2011). Diagnosing autism spectrum disorders in primary care. Practitioner.

[8] Achenbach, T. & Rescorla, L. Manual for the ASEBA preschool forms & profiles. *Univ. Vermont, Res. Cent. for Child. Youth & Fam*. (2000).

[9] Norris, M. & Lecavalier, L. Screening accuracy of level 2 autism spectrum disorder rating scales: A review of selected instruments. *Autism*, **14**, 263–284, 10.1177/1362361309348071 (2010). PMID: 20591956, 10.1177/1362361309348071 20591956

[10] Charman, T. & Gotham, K. Measurement issues: Screening and diagnostic instruments for autism spectrum disorders - lessons from research and practise. *Child Adolesc. Mental Heal*.**18**, 52–63, 10.1111/j.1475-3588.2012.00664.x. 10.1111/j.1475-3588.2012.00664.x PMC3607539 23539140

[11] Lord C, Rutter M, Le Couteur A (1994). Autism diagnostic interview-revised: a revised version of a diagnostic interview for caregivers of individuals with possible pervasive developmental disorders. J. Autism Dev. Disord..

[12] Lord C (1989). Autism diagnostic observation schedule: a standardized observation of communicative and social behavior. J Autism Dev Disord.

[13] Abbas, H., Garberson, F., Glover, E. & Wall, D. P. Machine learning approach for early detection of autism by combining questionnaire and home video screening. *J. Am. Med. Informatics Assoc*. ocy039, 10.1093_jamia_ocy039/1/ocy039 (2018). 10.1093/jamia/ocy039 PMC7646881 29741630

[14] Cognoa, Inc. 2390 El Camino Real St 220, Palo Alto, CA 94306 https://www.cognoa.com/.

[15] Wall, D. P., Dally, R. L., Luyster, R., Jung, J.-Y. & DeLuca, T. F. Use of artificial intelligence to shorten the behavioral diagnosis of autism. *PLoS One*, 10.1371/journal.pone.0043855. (2012). 10.1371/journal.pone.0043855 PMC3428277 22952789

[16] Lord C (2012). A multisite study of the clinical diagnosis of different autism spectrum disorders. Arch. Gen. Psychiatry.

[17] Kanne, S., Arnstein Carpenter, L. & Warren, Z. Screening in toddlers and preschoolers at risk for autism spectrum disorder: Evaluating a novel mobile-health screening tool. *Autism Res*. (2017). 10.1002/aur.1959 29734507

[18] Moody EJ (2017). Screening for autism with the srs and scq: Variations across demographic, developmental and behavioral factors in preschool children. J. Autism Dev. Disord..

[19] Aldridge, F. J., Gibbs, V. M., Schmidhofer, K. & Williams, M. Investigating the Clinical Usefulness of the Social Responsiveness Scale (SRS) in a Tertiary Level, Autism Spectrum Disorder Specific Assessment Clinic. *Journal of Autism and Developmental Disorders* **42**(8), 294–300 (2012). 10.1007/s10803-011-1242-9 21516433

[20] Schanding, G. T., Nowell, K. P. & Goin-Kochel, R. P. Utility of the Social Communication Questionnaire-Current and Social Responsiveness Scale as Teacher-Report Screening Tools for Autism Spectrum Disorders. *Journal of Autism and Developmental Disorders* **42**(8), 1705–1716 (2012). 10.1007/s10803-011-1412-9 22143742

[21] Jacobs RA (1995). Methods for combining experts’ probability assessments. Neural Computation.

[22] Falkmer T, Anderson K, Falkmer M, Horlin C (2013). Diagnostic procedures in autism spectrum disorders: a systematic literature review. Eur. Child & Adolesc. Psychiatry.

