# Supplementary material for: Multi-modular AI Approach to Streamline Autism Diagnosis in Young Children
Source: Sci Rep. 2020 Mar 19;10:5014. doi: 10.1038/s41598-020-61213-w (PMC7081341; doi:10.1038/s41598-020-61213-w)
Supplement: Supplementary file 1 — Supplementary Information. [file 41598_2020_61213_MOESM1_ESM.pdf]

Supplementary Material

Title: Multi-modular AI Approach to Streamline Autism Diagnosis in Young Children

Authors: Halim Abbas, Ford Garberson, Stuart Liu-Mayo, Eric Glover, Dennis P Wall

Supplementary Material

Time to Completion Comparisons

A random sample of 529 Cognoa users was used in order to measure time to completion of the parent module, including 358 corresponding to children under four years of age and 173 to children four years old and over. The median time to completion was just under 4 minutes, and the upper quartile of the range under 6 minutes.

For the clinician module, a sample of 232 assessment scorings were used, each completed during the child clinic visit, including 126 children under four years of age, and 106 children four and over. The median time to completion was 1 minute 11 seconds, and the upper quartile of the range under 2 minutes. Figure 5 shows time to completion distribution plots as well as breakdowns by age group for the parent and clinician modules.

The video assessment module doesn't take any time for the parent to complete beyond the shooting of 2 minutes of video on a mobile device. Video analysts then score those videos offline in order to render an assessment. For this module we collected self-reported turnaround time measurements from 6 video analysts over a total sample of 120 video assessment scorings, including 60 scorings corresponding to children under four years of age, and 60 for children four years old and over. The median turnaround time per analyst per video was under 20 minutes, and the upper quartile under 25 minutes. Figure 6 shows time to completion distribution plots as well as breakdowns by age group for the video analysis offline process.

Inconclusive Methodology

All classification algorithms used in this paper allow for three outcomes: positive, negative, and inconclusive. Figure 1 of the main document illustrates the training and application methodology of such algorithm. First, a preliminary binary classifier is trained and applied to the full training set. Cross validation is run repeatedly and a running tally of how often each sample was misclassified is recorded. This helps identify those samples which are harder for the Machine Learning model to predict correctly using the signal present in the dataset as a whole. Samples that are found to be classified incorrectly by the preliminary classifier at least half of the time were labeled as 'positive', and other samples were labeled as 'negative'. The preliminary classifier is then discarded, as it won't be used at inference time.

Next, the indeterminate classifier is trained on all the samples from the same training set, except that the target label used to train it is the 'positive' and 'negative' label from the preliminary classifier run. This indeterminate classifier will learn how to tell the difference between a hard-to-diagnose and easy-to-diagnose sample. Last, the autism classifier is trained only on easy-to-diagnose samples, and given the autism determination label as a target.

At inference time, the indeterminate classifier is applied first, and if it is positive, the sample is deemed inconclusive and no autism assessment is give. Only if it the indeterminate classifier output is negative is the sample deemed conclusive and the autism classifier applied to it for an autism assessment output.

Fully Conclusive Results

The assessment modules presented are intended to operate with an allowance for inconclusive determination at a tunable rate. The ROC curves in Figures 2 and 3 and the statistical significance results in Table 2 of the main document are based on a 30% inconclusive determination allowance.

For completeness, here we show the corresponding ROC curves on the entire validation sample, without the inconclusive advantage. Figure 7 shows the ROC curves over the full sample used in this clinical study, Figure 8 shows ROC curves for children under four years of age, and Table 3 shows the statistical significance comparison when our algorithms are forced to classify 100% of the hardest cases.

Differences Between the Training and Application Environments

Machine Learning models are trained using historical patient records that correspond to controlled, lengthy clinical examinations, but applied via web or mobile app aimed at unsupervised parents at home. Table 4 details the various mechanisms by which confounding biases may consequently creep into the application data. Biases of this nature can easily be significant enough to ruin the performance of an algorithm if not properly addressed, and cannot be probed by cross validation or similar analysis of the training data alone.

Our previous publication on this subject provides empirical evidence to the extent of the effect of these biases on the Cognoa assessment method, as well as measurements of progressive improvements gained by applying important counteractive machine learning techniques [13]. Major new strategies for addressing these biases are also introduced in this publication. For the parental questionnaire the question selection refinement resulting from studies of the bias led to an improvement in AUC (5% for young kids and 12% for older kids). For the video module these studies led to the injection of noise into the training dataset to compensate for this bias which resulted in an 11% improvement in the AUC.

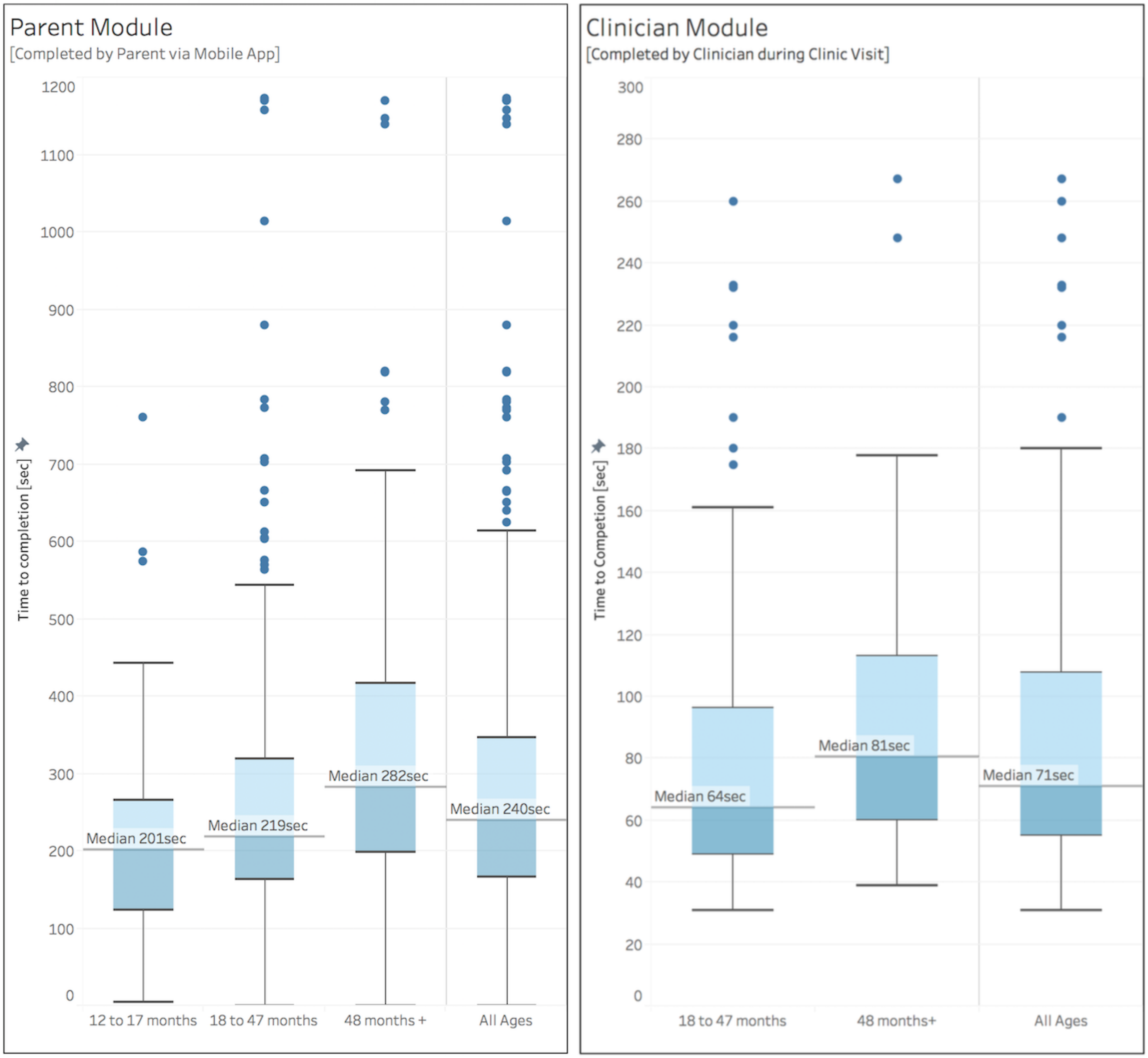

Figure 5. Time to completion for parent and clinician assessment modules, broken down by child age group.

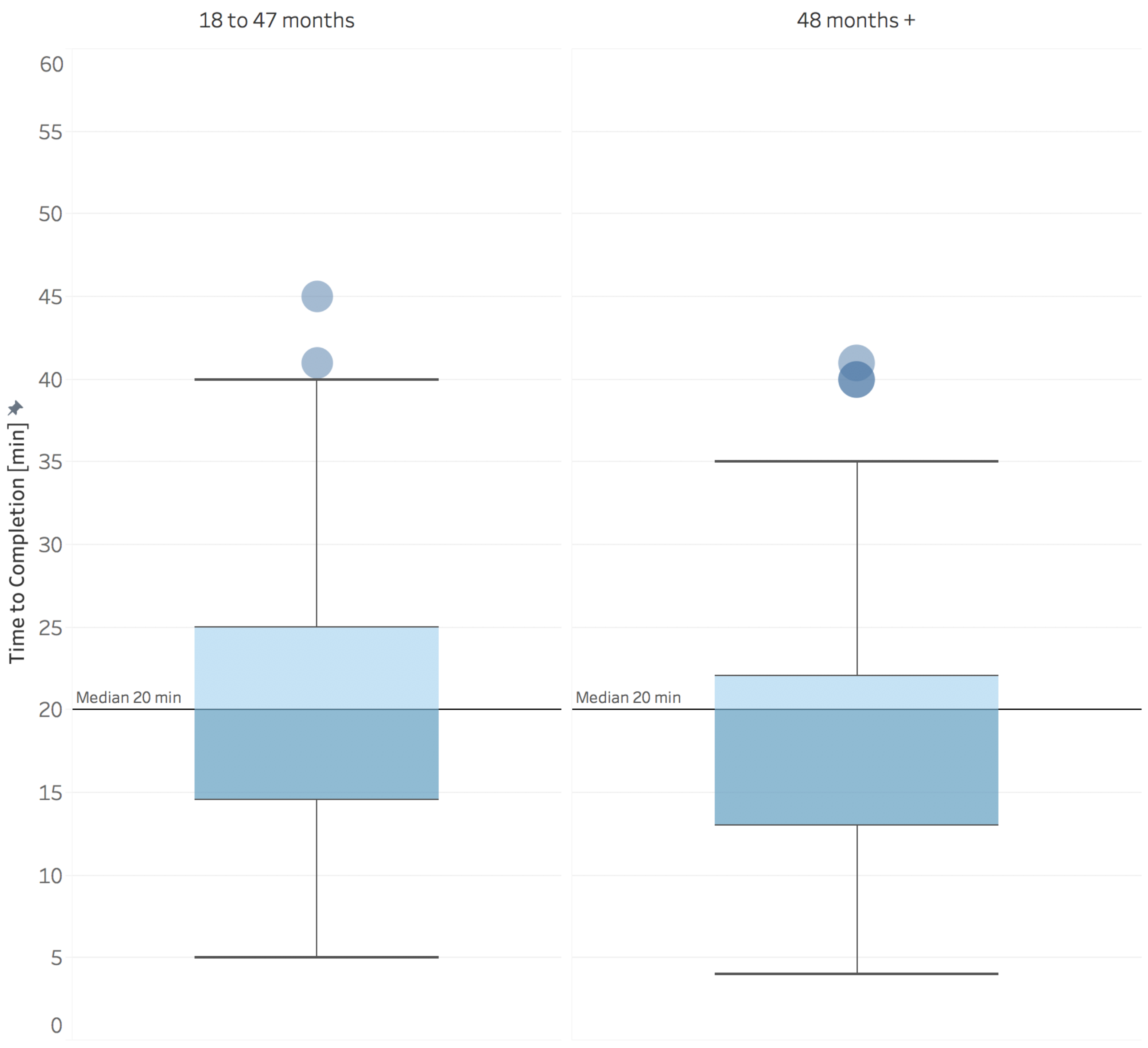

Figure 6. Time to completion for offline video analyst scoring process , broken down by child age group.

Table 3. Statistical tests of performance improvements between models in this paper and standard baseline screening **where the inconclusive methodology has been disabled**. See Table 2 of the main document for the same results when the algorithms are run with inconclusive outcomes allowed as intended. ΔAUC tells us the increase in AUC found in the screeners of this paper across bootstrapping experiments. ΔSpecificity tells us the increase in the specificity in the bootstrapping experiments at a threshold designed to achieve 90% sensitivity. Each Δ calculation shows the average value of the improvement along with the $[0.05, 0.95]$ confidence interval.

Age Group	Baseline Screener	Assessment Module	ΔAUC, [0.05, 0.95] C.I.	ΔSpecificity at 90% sensitivity, [0.05, 0.95] C.I.	*n*
All ages	CBCL	Parent	0.14, [0.09, 0.20]	0.17, [0.09, 0.24]	370
All ages	SRS-2	Parent	0.18, [0.11, 0.25]	0.15, [0.08, 0.23]	307
All ages	CBCL	Parent, Video	0.23, [0.17, 0.29]	0.20, [0.13, 0.28]	363
All ages	SRS-2	Parent, Video	0.26, [0.19, 0.33]	0.18, [0.10, 0.26]	302
All ages	CBCL	Parent, Video, and Clinician	0.31, [0.24, 0.39]	0.57, [0.47, 0.68]	200
All ages	SRS	Parent, Video, and Clinician	0.37, [0.29, 0.45]	0.53, [0.41, 0.65]	175
1.5 to 3 y.o.	MCHAT-R	Parent	-0.01, [-0.07, 0.06]	0.02, [-0.08, 0.13]	209
1.5 to 3 y.o.	CBCL	Parent	0.13, [0.04, 0.21]	0.12, [0.02, 0.23]	214
1.5 to 3 y.o.	SRS-2	Parent	0.15, [0.04, 0.25]	0.15, [0.05, 0.25]	161
1.5 to 3 y.o.	MCHAT-R	Parent, Video	0.09, [0.02, 0.15]	0.02, [-0.06, 0.10]	204
1.5 to 3 y.o.	CBCL	Parent, Video	0.23, [0.15, 0.32]	0.15, [0.05, 0.25]	209
1.5 to 3 y.o.	SRS-2	Parent, Video	0.24, [0.14, 0.34]	0.16, [0.06, 0.27]	157
1.5 to 3 y.o.	MCHAT-R	Parent, Video, and Clinician	0.14, [0.05, 0.23]	0.25, [0.07, 0.43]	107
1.5 to 3 y.o.	CBCL	Parent, Video, and Clinician	0.29, [0.19, 0.38]	0.40, [0.24, 0.57]	111
1.5 to 3 y.o.	SRS-2	Parent, Video, and Clinician	0.33, [0.22, 0.45]	0.44, [0.25, 0.64]	91
4 to 6 y.o.	CBCL	Parent	0.16, [0.08, 0.24]	0.21, [0.11, 0.31]	156
4 to 6 y.o.	SRS-2	Parent	0.21, [0.12, 0.30]	0.16, [0.06, 0.27]	146
4 to 6 y.o.	CBCL	Parent, Video	0.23, [0.15, 0.32]	0.26, [0.15, 0.37]	154
4 to 6 y.o.	SRS	Parent, Video	0.27, [0.17, 0.37]	0.20, [0.09, 0.32]	145
4 to 6 y.o.	CBCL	Parent, Video, and Clinician	0.34, [0.22, 0.45]	0.74, [0.61, 0.87]	89
4 to 6 y.o.	SRS-2	Parent, Video, and Clinician	0.40, [0.29, 0.51]	0.61, [0.46, 0.76]	84

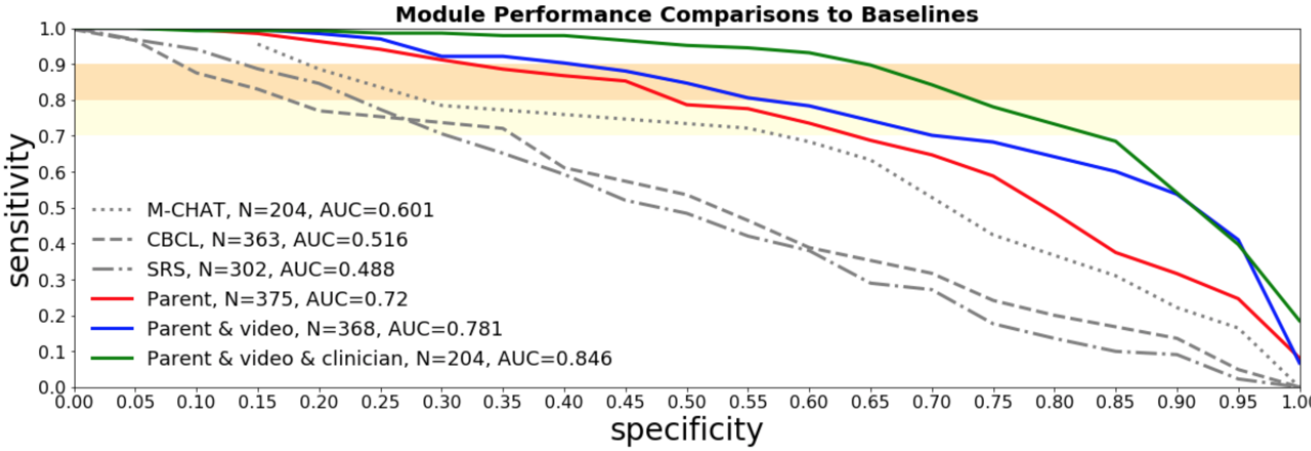

Figure 7. ROC curves on the clinical sample for the questionnaire, clinician and the video based algorithms, separately and in combination **where the inconclusive methodology has been disabled**. See Figure 2 of the main document for the same results when the algorithms are run with inconclusive outcomes allowed as intended. The established screening tools M-CHAT-R, CBCL, and SRS are included as baselines.

Hyperparameter Tuning Procedure

For each of the Machine Learning models underlying the parent, video, an clinician assessment modules, the following hyperparameters were optimized: the fraction of features considered at tree splits, the number of trees, the maximum depth of the trees, the learning rate of the gradient boosting algorithm, and the stochastic subsampling fraction.

The tuning was performed with the following procedure. For every possible combination of hyperparameters (within a reasonably chosen grid) a series of bootstrapping experiments was performed on the training dataset. In each bootstrapping experiment, class and age balancing was enforced using event weights, and then cross validation was run using a randomized set of 10 folds. The folds were each required to have equal proportions to each other of the autism and non-autism classes. During the training procedure, weights were applied in order to force equal contributions from both the autism and the non-autism classes and for each age in the training folds. The AUC metric was calculated on the results from the validation folds with weighting to force equal contribution from each age group, and the average of the AUC values across all bootstrapping experiments was used to determine the best combination of hyperparameters. The above procedure was found to lead to much more consistent results than simply using cross validation to determine the optimal hyperparameter combination, which can lead to AUC results that fluctuate by 2-3% depending on the folds chosen or the randomness of the training procedure.

Models were eventually retrained on the optimally chosen hyperparameters, and the clinical studies discussed in this paper are relied upon for final performance validation.

Potential for Module Stacking to Improve Clinical Workflow

Progressive sampling runs were performed to assert available training data is sufficient to build stable Machine Learning classifiers. These runs were performed for each classifier variant for both the guardian and the video based training modules. In each run, boot-strapped cross-validation was used to compute the AUC metric of the algorithm trained over increasingly larger proportions of the training set. The size of the training set was demonstrated to be sufficient for stable learning of ensemble decision trees, as shown in the plots of Figure 9 (for the guardian module) and Figure 10 (for the video module). It can be seen that, while having more data would always be beneficial, for all screening scenarios the sample sizes are large enough to achieve good performance.

Progressive Application of Diagnostic Modules

As an alternative to running each of the diagnostic modules on each patient, here we simulate the effect of selective progression in a sequence of modules with termination decision on a case by case basis. For every patient, we start with the parent questionnaire module. If that gives a negative determination we stop, otherwise we proceed with the video module and combine both outputs into a single outcome. If that in turn gives a negative determination we stop, otherwise we proceed with the clinician module and combine all three outputs into a single final outcome, whether conclusive or not.

The advantage of this arrangement is mainly practicality in real life application, where some parents would benefit from a reliable early negative determination without having to undergo one or two additional modules. The concern of course is degradation in overall accuracy, since some of the patients will be given a determination absent the information from some of

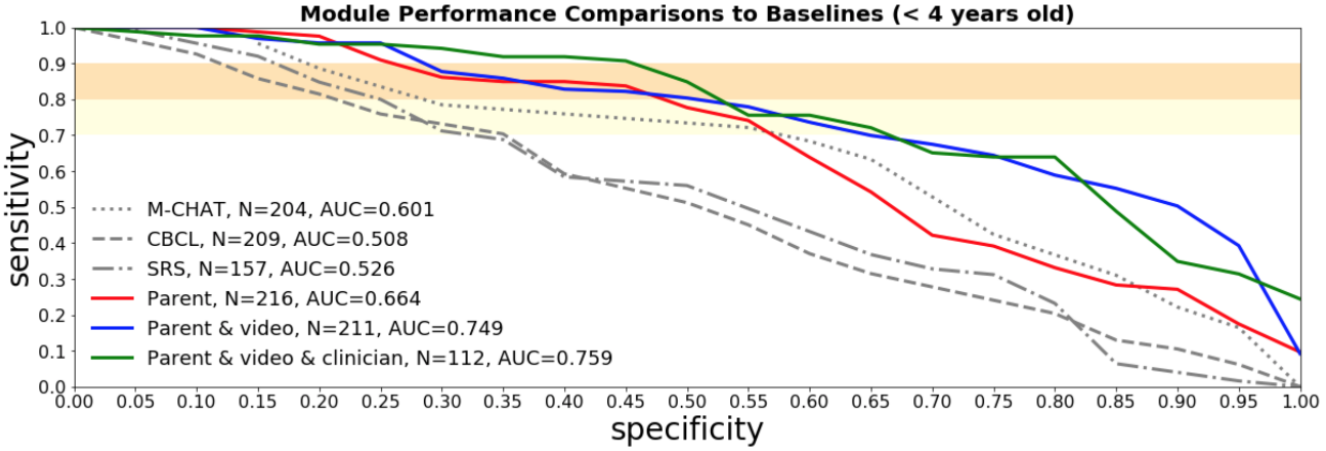

Figure 8. ROC curves on kids < 4 years of age in the clinical sample for the questionnaire, clinician and the video based algorithms, separately and in combination **where the inconclusive methodology has been disabled**. See Figure 3 of the main document for the same results when the algorithms are run with inconclusive outcomes allowed as intended. The established screening tools M-CHAT-R and CBCL are included as baselines.

the later stage modules.

In order to assess the overall reliability of this progressive diagnostic setup, we used the clinical study dataset to tune and simulate it. The results are shown in Figure 11, including accuracy metrics for the overall system as well as for the individual stages in the progression.

Having tuned and simulated progressive screening on the same dataset leaves an open concern regarding over-fitting. Future work is needed to assess how well these results would generalize on prospective data. However from this experiment, it does seem likely that such progressive screening can be achieved with reliable overall accuracy.

Table 4. Differences between training and application environments. These differences are expected to cause bias that cannot be captured by cross validation studies.

	Training Setting	Application Setting
Source	Standardized instrument administered by trained professionals during clinical evaluations	Short parent questionnaires displayed on smartphone, and behavior tagging by analysts after observing two or three 1-minute home videos uploaded by parents
Proctor	Custom trained medical professionals	Parents answering the questionnaires are untrained, and the analysts evaluating the home videos are only minimally trained. As a result, their answers may not be as consistent, objective, or reliable
Setting	Clinic setting with standardized and semi-structured interactions	At home. Not possible to recreate the structured clinical environment, resulting in an undesired variability of the output signals. Subjects might also behave differently at the clinic than at home, further amplifying the bias
Duration	The ADI-R takes 90-150 minutes to complete; The ADOS 40-60 minutes of direct observation by trained professionals	Under 10 minutes to complete the parent questionnaire, and a few minutes of home video. As a result, some symptoms and behavioral patterns might be present but not observed. Also causes big uncertainty about the severity and frequency of observed symptoms
Questionnaires	Sophisticated language involving psychological concepts, terms, and subtleties unfamiliar to non experts	Simplified questions and answer choices result in less nuanced, noisier inputs

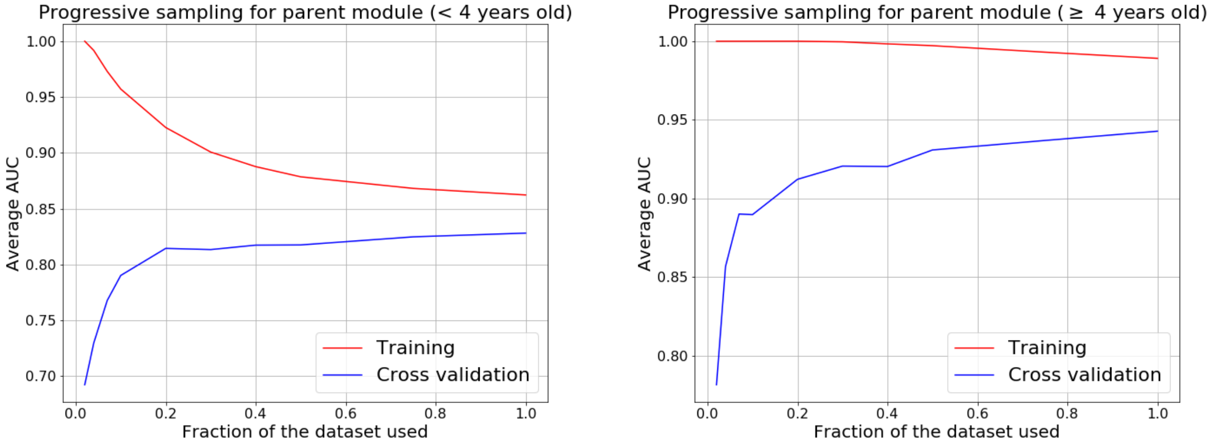

(a) Children < 4 years old

(b) Children ≥ 4 years old

Figure 9. Progressive sampling used to establish the availability of sufficient training data for training a Machine Learning classifier for the guardian assessment module. The AUC is shown without making use of the inconclusive technique.

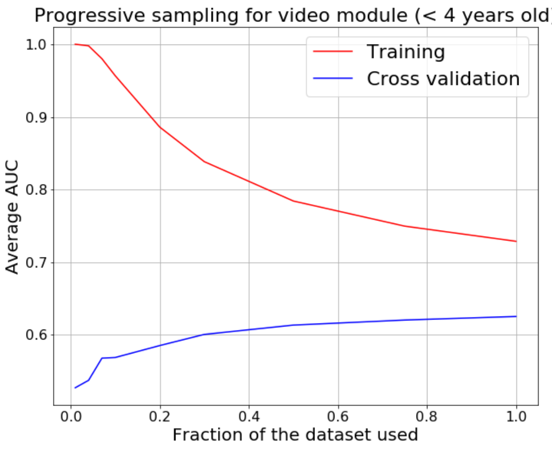

(a) Children $<$ 4 years old

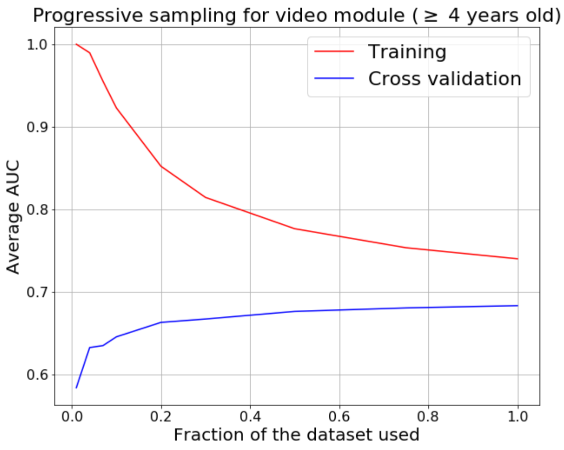

(b) Children $\geq$ 4 years old

Figure 10. Progressive sampling used to establish the availability of sufficient training data for training a Machine Learning classifier for the video assessment module. The AUC is shown without making use of the inconclusive technique.

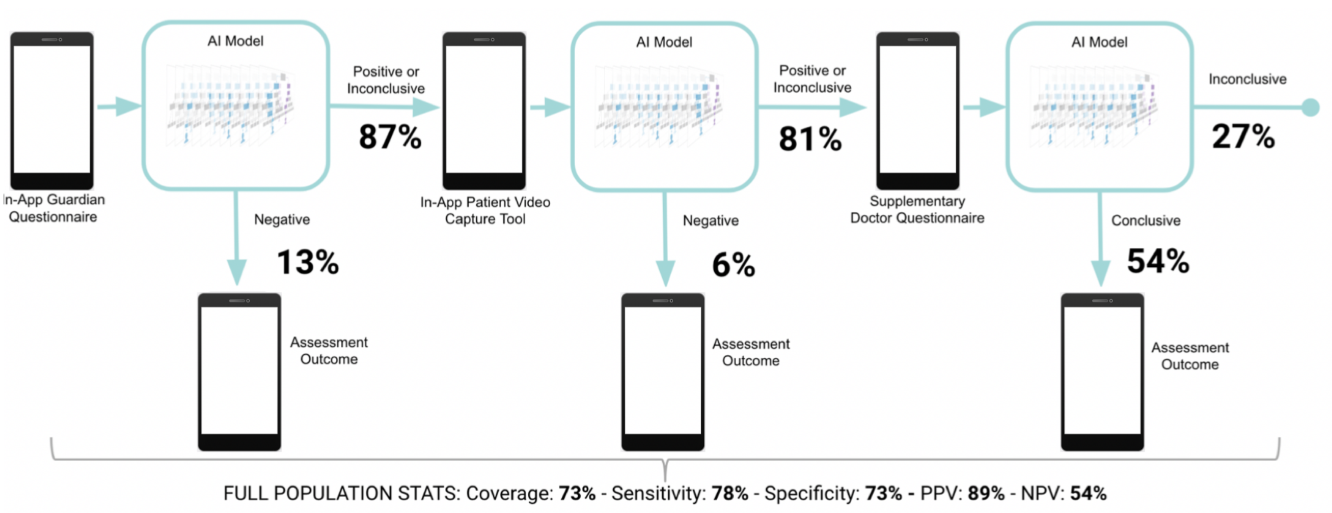

Figure 11. Progressive diagnosis simulation setup and accuracy results
